# Ethanolic Extract of *Averrhoa carambola* Leaf Has an Anticancer Activity on Triple-Negative Breast Cancer Cells: An In Vitro Study

**DOI:** 10.3390/pharmaceutics17010002

**Published:** 2024-12-24

**Authors:** Oscar F. Beas-Guzmán, Ariana Cabrera-Licona, Gustavo A. Hernández-Fuentes, Silvia G. Ceballos-Magaña, José Guzmán-Esquivel, Luis De-León-Zaragoza, Mario Ramírez-Flores, Janet Diaz-Martinez, Idalia Garza-Veloz, Margarita L. Martínez-Fierro, Iram P. Rodríguez-Sanchez, Gabriel Ceja-Espíritu, Carmen Meza-Robles, Víctor H. Cervantes-Kardasch, Iván Delgado-Enciso

**Affiliations:** 1 Department of Molecular Medicine, School of Medicine, University of Colima, Colima 28040, Mexico; oscar.beas.11@gmail.com (O.F.B.-G.); gahfuentes@gmail.com (G.A.H.-F.); mario_ramirez@ucol.mx (M.R.-F.); gcejae11@ucol.mx (G.C.-E.); vkardasch@ucol.mx (V.H.C.-K.); 2 State Cancerology Institute of Colima, Health Services of the Mexican Social Security Institute for Welfare (IMSS-BIENESTAR), Colima 28085, Mexico; arianacabrera267@gmail.com (A.C.-L.); drluisdz@gmail.com (L.D.-L.-Z.); carmen.qfb@gmail.com (C.M.-R.); 3 Faculty of Chemical Sciences, University of Colima, Coquimatlan 28400, Mexico; 4 Faculty of Sciences, University of Colima, Colima 28045, Mexico; silvia_ceballos@ucol.mx; 5 Clinical Epidemiology Research Unit, Mexican Institute of Social Security, Villa de Alvarez, Colima 28984, Mexico; jose.esquivel@imss.gob.mx; 6 Research Center in Minority Institutions, Florida International University (FIU-RCMI), Miami, FL 33199, USA; jdimarti@fiu.edu; 7 Molecular Medicine Laboratory, Academic Unit of Human Medicine and Health Sciences, Autonomous University of Zacatecas, Zacatecas 98160, Mexico; idaliagv@uaz.edu.mx (I.G.-V.); margaritamf@uaz.edu.mx (M.L.M.-F.); 8 Molecular and Structural Physiology Laboratory, School of Biological Sciences, Autonomous University of Nuevo Leon, San Nicolas de los Garza 66455, Mexico; iramrodriguez@gmail.com; 9 Robert Stempel College of Public Health and Social Work, Florida International University, Miami, FL 33199, USA

**Keywords:** *Averrhoa carambola*, breast cancer, triple-negative breast cancer, MDA-MB-231 cells, polyphenols, flavonoids, traditional medicine

## Abstract

**Background/Objectives**: *Averrhoa carambola*, or star fruit, is a shrub known for its medicinal properties, especially due to bioactive metabolites identified in its roots and fruit with anti-cancer activity. However, the biological effects of its leaves remain unexplored. This study aimed to assess the effects of ethanolic extract from *A. carambola* leaves on triple-negative breast cancer (TNBC), an aggressive subtype lacking specific therapy. **Methods**: Phytochemical analysis and HPLC profile and additional cell line evaluation employing MDA-MB-231 were carried out. **Results**: Phytochemical screening revealed that the ethanolic extract was rich in flavonoids, saponins, and steroids, demonstrating an antioxidant capacity of 45%. ^1^H NMR analysis indicated the presence of flavonoids, terpenes, and glycoside-like compounds. Cell viability assays showed a concentration-dependent decrease in viability, with an IC_50_ value of 20.89 μg/mL at 48 h. Clonogenic assays indicated significant inhibition of replicative immortality, with only 2.63% survival at 15 μg/mL. Migration, assessed through a wound healing assay, was reduced to 3.06% at 100 μg/mL, with only 16.23% of cells remaining attached. An additive effect was observed when combining lower concentrations of the extract with doxorubicin, indicating potential synergy. **Conclusions**: These results suggest that the ethanolic extract of *A. carambola* leaves contains metabolites with anti-cancer activity against TNBC cells, supporting further research into their bioactive compounds and therapeutic potential.

## 1. Introduction

Breast cancer is one of the most common cancers diagnosed in women worldwide [1]. Statistics from 2022 revealed that it was the second most commonly diagnosed cancer, with an estimated 2.29 million new cases, equivalent to 11.5% of all cancers or 1 in 4 cases [1]. It also ranks as the fifth leading cause of cancer-related deaths, with 666,103 deaths worldwide, accounting for one in six cancer deaths [1]. Triple-negative breast cancer (TNBC) is a particularly aggressive subtype of breast cancer, characterized by the absence of estrogen and progesterone receptors and the lack of HER2 protein expression [2,3]. Epidemiological data show that TNBC represents approximately 15–20% of all types of breast cancer [4]. Compared to other breast cancer subtypes, women diagnosed with TNBC are 53% more likely to be under the age of 40 [5,6]. Treatment of this type of cancer is a challenge as there is no biological target against which specific therapies can be employed, and its heterogeneous intertumoral and intratumor molecular complexity is such that it is further classified into six molecular subtypes [7,8]. Recently, the androgen receptor (AR), the epidermal growth factor receptor (EGFR), and the tumor suppressor gene BRCA1 have been postulated as possible biomarkers; however, its potential as a therapy target is still under study [8,9,10,11,12,13]. Surgery remains the primary treatment approach; however, recurrence occurs in approximately 25% of cases, with an average relapse period of 19 to 40 months and a 75% mortality rate within the first three months after recurrence [14,15]. Radiotherapy and chemotherapy are neoadjuvant treatments; however, only about 30–45% of patients respond successfully in the non-metastatic stage [15,16]. Furthermore, these treatments can be aggressive and have significant side effects, with a median survival time after metastasis of 13.3 months [15,16].

Anthracyclines are one of the first lines of chemotherapeutics used against this type of cancer [17,18]. Doxorubicin belongs to this family and is commonly used in the clinical approach because of its high response rate in metastatic TNBC [19]. Unfortunately, it causes a range of side effects, including neurotoxicity [20], as well as common adverse reactions associated with other chemotherapeutic agents, such as nausea, vomiting, gastrointestinal disturbances, and alopecia. Moreover, it can result in long-term complications, particularly cardiotoxicity and gonadotoxicity [20,21,22]. Another disadvantage is intrinsic resistance and/or the development of resistance [23]. In this context, efforts to treat TNBC focus on the typing of subtypes to establish biological targets and, consequently, on the development of targeted therapies, which involves the search for, or the design of drugs against these targets. One possible source of new pharmacological agents may be the bioactive metabolites of plants, specifically those commonly used in traditional medicine [24,25].

*A. carambola*, commonly known as star fruit, is a perennial tropical shrub from the family *Oxalidaceae* [26]. Native to Southeast Asia, it has become prevalent in the tropical and subtropical regions of the Americas [26]. Various parts of the tree, including leaves, roots, fruit, and flowers, are used in traditional Chinese, Indian, Malay, and Brazilian medicine [26,27]. For example, crushed leaves are used as a topical treatment for chickenpox, ringworm, and headaches. Its infusions are used to treat stomach pain, fever, aphthous stomatitis, tonsillitis, high blood pressure, diabetes mellitus, and urinary system diseases [27,28,29]. Regarding anti-cancer activity, the compound 2-didecyl-6-methoxylcyclohexa-2,5-dien-1,4-dione (DMDD), isolated from the roots, showed an apoptotic selective effect against breast cancer cell lines MCF-7, BT20, and MDA-MB-231, human lung carcinoma line A549, and osteosarcoma cell line U2OS [26,30]. Their mechanisms of action involve caspase-8 activation, TRAIL-R1, and TRAIL-R2 death receptors upregulation, cytochrome c release, and NF-kB inhibition [30,31,32]. Also, it has been reported that the ethanolic extract of the fruit exhibits antitumor activity in a murine model of liver cancer, through the increased in GSH, SOD, and CAT enzymes and lipid peroxidation inhibition [33,34]. Considering the available information on the phytochemistry of *A. carambola*, previous preclinical evaluations on diabetes [35,36,37], and the identification of some compounds in the leaf, roots, and fruit, along with the existing gap in knowledge regarding the evaluation of this plant species against triple-negative breast cancer, studying this plant becomes crucial given the significance of this cancer type and the potential for discovering novel therapeutic approaches.

Therefore, this study aims to evaluate the effects of an ethanolic extract of *A. carambola* leaves on triple-negative breast cancer in vitro. Firstly, a preliminary screening analysis of the phytochemical characteristics of the ethanol extract was carried out employing spectroscopic and antioxidant tests. Subsequently, its impact on some of the hallmarks of cancer was studied. Using the MDA-MB-231 cell line, representative of the mesenchymal stem-like subtype, its inhibitory activity on viability, proliferation, migration, and adhesion of these cells was established. Finally, the outcome of combining low doses of the extract with low doses of doxorubicin, one-fifth of inhibitory concentration 50 (IC_50_), on cell viability was examined and it was observed that the mixture reduced cell viability similarly to the IC_50_ of doxorubicin. These results strongly suggest that the leaf extract may contain bioactive metabolites with inhibitory activity against this type of cancer cell. Further analysis is therefore warranted to systematically characterize the compounds and to clearly establish whether they can be used as adjuvant therapies against triple-negative breast cancer.

## 2. Materials and Methods

### 2.1. Averrhoa carambola Leaf Ethanolic Extract Preparation

*Averrhoa carambola* leaves were collected in the municipality of Comala, Colima state, Mexico (19.330430 N, −103.747084 W), at an altitude between 500 and 3800 m. The collection took place in March 2023 under a warm subhumid climate with summer rains and lower humidity (38.64%). The collection site is characterized by Regosol soil type and vegetation predominantly of tropical forest. The species was taxonomically identified by comparing it with a previously deposited herbarium specimen from the National Herbarium of Mexico (MEXU), catalog number 1053019. The leaves were meticulously cleaned, dried at 38 °C, and finely ground into a powder using an industrial blender model LM-12 12 L, (insumos & equipos JR^MR^, Cartagene, Colombia) in 60 s pulses. Five hundred grams of the powdered material underwent cold maceration for 24 h in 2L of 96% ethanol (AZ, Jalisco, Mexico) with stirring, at room temperature, using a rotary shaker (HZ-300, JIN YI^®^, Jiangsu, China). The resulting mixture was filtered through 2.8 µm porosity filter paper (Whatman^®^ Cytiva, Marlborough, MA, USA) and was concentrated for 6 h using a rotary evaporator HB10 (IKA^®^-Werke, Germany ) at low pressure at 37 °C. The residues were reunited and weighed, and the yield was determined (%*w*/*w*). The ethanolic extract was stored at −10 °C under N_2_ atmospheric conditions, adhering to the protocol outlined by Hernández-Fuentes et al., 2019 [38].

### 2.2. Qualitative Phytochemical Analysis of A. carambola Leaf Extract

The methodology outlined by Oloya et al. (2021) was used [39]. Tannin levels were assessed using a saturated ferric chloride solution. Flavonoids were determined using concentrated HCl and magnesium, along with the Marini Bettolo test, which involves a solution of SbCl_5_ in CCl_4_, taking into account the phytochemical profile of the species’ flavonoid variants. Alkaloids were identified using the Dragendorff, Mayer, and Wagner tests, while saponins were evaluated through the hemolysis test with 7% blood agar and foam formation. The presence of steroid rings was detected by their reaction with H_2_SO_4_ in a chloroform solution. All analyses were carried out using a stock solution of the extract at a concentration of 0.1 mg/mL in methanol [38].

### 2.3. Spectroscopic Profile of the A. carambola Leaf Extract

UV data of the extract stock at 0.1 mg/mL in methanol were collected employing a spectrophotometer (Evolution 300) in MeOH solution. FTIR spectroscopic data were acquired using a Varian 660-IR spectrophotometer. A preliminary NMR profile of the extract was conducted using an NMR spectrometer (Bruker, Leipzig, Germany) operating at a frequency of 400 MHz in dimethyl sulfoxide-d_6_, DMSO-d_6_, (Sigma-Aldrich, Saint Louis, MO, USA) served as the solvent for the extract. Chemical shifts were measured in δ (ppm) and coupling constants (*J*) were reported in *Hz* [40]. The chemical shifts obtained were compared with those of some isolated compounds from *A. carambola* leaves [26,28,37,41] (see Supplementary Table S1).

### 2.4. Total Flavonoid Content in A. carambola Leaf Extract

The methodology described by Chang et al. (2020), with adaptations from Wakeel et al. (2019), was used [42,43]. Briefly, dilutions of *A. carambola* ethanolic extract were prepared from a 0.1 mg/mL stock solution in methanol, resulting in a final volume of 40 µL. To this, 20 µL of 10% AlCl_3_, 20 µL of CH_3_CO_2_K 1M, and 380 µL of H_2_O_2_ were added. The reaction mixture was incubated for 30 min and then measured at a wavelength of 405 nm employing a spectrophotometer BioMate3 (BioMate^TM^, Thermo, Madison, WI, USA). A standard curve was created with quercetin concentrations of 50, 25, 12.5, and 6.25 µg/mL. The employed curve was the following (y = 0.0029x + 0.0057, R2 = 0.9996). Total flavonoid content was quantified and expressed as quercetin equivalents in µg/mg of the *A. carambola* extract.

### 2.5. Total Antioxidant and Reductive Capacity of A. carambola Leaf Extract

The phosphomolybdenum assay was performed following an adapted protocol [44,45]. In summary, 20 µL of the *A. carambola* ethanolic extract (0.1 mg/mL in methanol) was combined with 180 µL of a phosphomolybdenum reagent containing 0.6 M H_2_SO_4_, 28 nM NaH_2_PO_4_, and 4 mM (NH₄)₆Mo₇O₂₄. The mixture was incubated at 95 °C for 90 min in a water bath, after which the absorbance was measured at 630 nm using a spectrophotometer BioMate3 (BioMate^TM^, Thermo, Madison, WI, USA). Ascorbic acid was used as the positive control, and the antioxidant capacity was calculated using the following formula: % Antioxidant capacity = [1 − (OD sample/OD control)] * 100.

The reducing power of the ethanolic extract was evaluated using a modified version of the potassium ferrocyanide–ferric chloride method, as described by Wakeel et al. (2019) [43]. The analysis was performed mixing 40 µL of the ethanolic extract stock at 0.1 mg/mL in methanol with 50 µL of phosphate buffer (0.2 M, pH 6.6) and 50 µL of 1% K₄[Fe(CN)₆], followed by incubation at 50 °C for 20 min. Subsequently, 50 µL of 10% CCl₃COOH was incorporated, finally, the mixture was centrifuged at 3000 rpm for 10 min. The layer on the top was collected, and 33.3 µL of 0.1% FeCl_3_ was added. The samples were measured using a spectrophotometer BioMate3 (BioMate^TM^, Thermo, Madison, WI, USA), at a wavelength of 630 nm. The reducing power was determined using the following formula: % Reducing power = [1 − (OD sample/OD control)] * 100.

### 2.6. Polyphenol Content by Folin–Ciocalteu Method

The total phenolic content (TPC) was measured using the Folin–Ciocalteu assay as described by Kähkönen et al. (1999) with modifications based on the method reported by Chang et al., 2010 [46,47,48]. For the analysis, triplicate samples (300 µL each) were placed into test tubes, and then 1.5 mL of 10-fold diluted Folin–Ciocalteu reagent and 1.2 mL of 7.5% (*w*/*v*) sodium carbonate solution were added. The mixture was allowed to stand for 30 min before measuring absorbance at 765 nm. The total phenolic content (TPC) was expressed as gallic acid equivalents (GAE) in mg per 100 g of material. The calibration curve for gallic acid was calculated using the equation y = 0.4994x − 0.4825 (R^2^ = 0.9985), where y is the absorbance and x is the gallic acid concentration in mg/L. The same procedure was applied to *A. carambola* samples, which were tested at a concentration of 0.5 mg/mL for both the extract and hydrolysate of the leaves (ethanol/water) [44,46,47,48].

### 2.7. DPPH Scavenging Activity from Extract of A. carambola

The DPPH scavenging activity of *A. carambola* was evaluated using a modified protocol based on the method by Lee K et al., 2015 [49], with ascorbic acid (Sigma-Aldrich, Mexico) serving as the control. A 0.004% DPPH-ethanol solution was prepared and stored at 4 °C. Different extract concentrations (ranging from 0.005 to 2 mg/mL) were prepared. Then, 1 mL of each sample solution was mixed with 1 mL of the prepared DPPH solution and 2 mL of anhydrous ethanol. The reaction mixture was kept in the dark for 30 min to allow the reaction to occur. Absorbance was measured at 517 nm, and the scavenging activity (Ax) was calculated. Distilled water was used as the blank (A0), and the control was represented by ascorbic acid (Ay). The scavenging activity was calculated using the equation: Scavenging activity (%) = [A0 − (Ax − Ay)] / A0 * 100% [47,50,51].

### 2.8. Chromatographic Analysis by HPLC

The chromatographic profile of *A. carambola* extract and hydrolysate of the leaves was determined using a polyphenol-based analytical approach by adapting the methodologies of [52], Sakakibara (2003), V. Cijo George (2015) [53], and Hernández-Fuentes (2020) [54]. A Waters e2695-Alliance system equipped with a photodiode array detector (DAD, model 2998, Waters Corporation, Milford, MA, USA) was used. Compound separation was achieved using an XBRIDGE C18 column of 150 mm × 4.6 mm and particle size of 3.5 µm (Waters Corporation, Milford, MA, USA).

The mobile phase consisted of acetonitrile and water acidified with 0.05% formic acid, with a gradient program designed to optimize compound separation. The gradient started with 20% acetonitrile and 80% acidified water for the first 10 min, gradually increasing to 80% acetonitrile by 40 min, and maintaining this ratio until 50 min. The initial ratio was restored between 50 and 60 min. The flow rate was set at 0.8 mL/min, with an injection volume of 20 μL, and the column temperature maintained at 35 ± 5 °C. UV–VIS spectral data were recorded in the 200–400 nm range. The total analysis runtime was 60 min. Reference standards used included gallic acid (GA), cinnamic acid (CA), anthrone (ANT), quercetin (Q), and 4-methylumbelliferone (4-ML) (all the standards were purchased by Sigma-Aldrich, USA). The standards used for HPLC were chosen based on their relevance to the identification of phenolic compounds in *A. carambola* extract. They included common phenolic acids such as gallic acid (GA), a phenolic acid; flavonoids like quercetin (Q), a flavonoid; as well as other compounds like anthrone (ANT), a flavanone, cinnamic acid (CA), a phenolic acid, and 4-methylumbelliferone (4-ML), a coumarin [55]. These standards were selected as they represent the types of compounds expected to be present in the extract, allowing for the comparison of retention times and the identification of similar compounds in the chromatograms of the plant extract [56]. All reagents used, including HPLC-grade acetonitrile and milliQ water (Sigma-Aldrich, Saint Louis, MO, USA), were prepared for the mobile phase, samples, and standards. Samples were filtered prior to injection using 0.22 µm pore size syringe Filters (TPP®, Trasadingen, Switzerland). Weighing measurements were performed using a high-precision analytical balance (Explorer™ Precision, Ohaus Corporation, Parsippany, NJ, USA).

For sample preparation, both the standards and the extract were diluted in a mixture of acetonitrile–acidified water (90:10), with the acidified water prepared using water and 0.05% formic acid. The samples and standards were injected immediately after dilution. To ensure repeatability, multiple injections were performed with concentrations ranging from 100 to 1000 ppm for the standards and 500–5000 ppm (parts per million), for the extract and hydrolysate of the leaves. Standards were first injected individually, and then a mixture of five standards in a 1:1 ratio was prepared and injected in the same acetonitrile–acidified water mixture (90:10).

The hydrolysate was prepared according to the methodology of Ross et al. (2009) [57], using 0.5 g of *A. carambola* leaves and treated with a mixture of 10 mL of HCl (12 N), incubated with heating at 80 °C for 30 min. Subsequently, 15 mL of chloroform (Sigma-Aldrich, Saint Louis, MO, USA) was added, and this process was repeated three times. The organic phases were combined, dried with anhydrous sodium sulfate (Merck, Darmstadt, Germany), and concentrated under reduced pressure using a rotary evaporator. The residues were weighed and stored in an amber bottle under inert atmosphere until further analysis.

### 2.9. Preparation of A. carambola Leaf Extract Treatments

The ethanolic extract of *A. carambola* (10 mg) was dissolved in 1 mL of dimethyl sulfoxide (DMSO) (Sigma-Aldrich, Saint Louis, MO, USA) and shaken at room temperature. A dilution at 1 mg/mL was prepared in Dulbecco’s Modified Eagle’s (DMEM/F-12, Biowest®, Bradenton, FL, USA) supplemented with 1X penicillin/streptomycin (Antibiotic-Antimycotic 100X, Gibco^TM^, Thermo Fisher, Grand Island, NY, USA). The concentrations to be evaluated of 75, 100, 125, 150, and 200 μg/mL were prepared in DMEM/F-12 supplemented with 1% (*v*/*v*) fetal bovine serum (FBS, Biowest®, Bradenton, FL, USA) and 1X penicillin/streptomycin. Doxorubicin (Teva Pharma AG, Basel, Switzerland) was used at an IC_50_ dose for 48 h of 2 μM (1.159 mg/mL) [58,59]. DMEM-F12 medium containing 0.1% DMSO was used as the negative damage control, while DMEM/F-12 medium supplemented with 1% FBS served as the blank control.

### 2.10. Cell Lines

The MDA-MB-231 cell line from human triple-negative breast cancer (ATCC: HTB-26TM, Manassas, VA, USA) and the TC-1 cell line from murine cervical cancer (ATCC: CRL-2493; Manassas, VA, USA) were maintained in DMEM/F-12 (Biowest®, Bradenton, FL, USA) supplemented with 10% (*v*/*v*) FBS (Biowest®, Bradenton, FL, USA) and 1X penicillin/streptomycin (Antibiotic-Antimycotic 100X, Gibco^TM^, Thermo Fisher, Grand Island, NY, USA ). Cells were incubated in a humidified incubator with 5% CO_2_ at 37 °C in 95% air. For cell harvesting during subculturing, 0.25% Trypsin–EDTA Solution (Gibco^TM^, Thermo Fisher, Grand Island, NY, USA) was used.

### 2.11. Cell Viability Assay/Cytotoxicity

The cell lines at a density of 1 × 10^4^ cells/100 µL/well were seeded in a 96-well TC-treated flat-bottom microplate (TPP®, Trasadingen, Switzerland) in DMEM/F-12 medium supplemented with 10% (*v*/*v*) FBS and 1X penicillin/streptomycin and incubated for 24 h in a humidified incubator at 37 °C in 95% air and 5% CO_2_. After, the medium was replaced with 100 μL/well of previously described treatments (Section 2.9) and incubated in the same conditions for 48 h. Finally, treatments were replaced with 100 μL/well of 1*X* Alamar Blue TM cell viability assay reagent (Sigma-Aldrich, Saint Louis, MO, USA) in a serum-free medium and incubated for 4 h. Microplates were read at 570 nm excitation and 600 nm emission wavelengths in a microplate reader (iMark^TM^, Bio-Rad, Hercules, CA, USA). The cytotoxicity percentage was calculated as %Cytotoxicity = 100 − [(experimental OD value − blank OD value)/(control OD value − blank OD value) × 100%]. To calculate the IC_50_ value, a nonlinear regression curve fit analysis was applied [58]. Images of morphological changes were captured with the inverted microscope AE31 EPI coupled to the Moticam Pro 252A camera and Motic Images Plus 2 software (Motic^®^, Xiamen, China). 

### 2.12. Clonogenic Assay

MDA-MB-231 cells were seeded and treated as described in Section 2.11. Cells were cultured for 48 h at 37 °C in a 5% CO_2_ atmosphere. Following treatment, the culture medium was removed, and the cells were washed with warm, sterile 1*x* PBS (pH 7.4). The cells were then detached using 0.25% trypsin–EDTA (Gibco^TM^, Thermo Fisher, Grand Island, NY, USA), stained with trypan blue (Sigma-Aldrich, Saint Louis, MO, USA), and counted. For each treatment, 1 × 10^3^ cells were plated into a TC-treated 24-well plate containing 500 μL of DMEM/F-12 medium supplemented with 10% FBS and 1X penicillin/streptomycin. The plates were incubated for 14 days. At the end of the incubation period, the cells were fixed with buffered formalin for 15 min, stained with 0.4% crystal violet for 45 min, and rinsed with water to remove excess stain. Images were captured [60], and colonies were counted. The survival percentage was determined using the following formula: (number of colonies in treatment × 100)/number of colonies in control.

### 2.13. Wound Healing Assay 

Monolayers of MDA-MB-231 cells at approximately 90% confluence were scraped using a 200 μL tip and subsequently washed with warm sterile 1X PBS (pH 7.4) to remove non-adherent cells. The cells were treated with the ethanolic extract concentrations as outlined in Section 2.9, with the modification that the medium was serum free and were incubated for 48 h [61]. Images were obtained at 0 and 48 h using the AE31 EPI inverted microscope equipped with a Moticam Pro 252A camera and analyzed with Motic Images Plus 2 software with a 4× magnification (Motic^®^, Xiamen, China). The captured images were further processed using TScratch software (www.cse-lab.ethz.ch/software.html (accessed on 20 December 2024)) [62] to calculate the percentage of area reduction using the following formula: 100 − [(areaTX × 100)/area] T0, where Tx = the area at 48 h, and T0 represents the area at the start of the experiment.

### 2.14. Adhesion Assay

In a TC-treated 24-well plate, MDA-MB-231 cells were seeded at a density of 2 × 10^4^ cells/500 µL/well in DMEM/F-12 medium supplemented with 10% (*v*/*v*) FBS and 1X penicillin/streptomycin and incubated for 24 h in a humidified incubator at 37 °C in 95% air and 5% CO_2_. After, the medium was replaced with 500 µL/well of previously described treatments (Section 2.9) and incubated for 48 h. In the end, on the one hand, the unattached cells present in the medium were recovered stained with trypan blue, counted, and re-seeded in a TC-treated 24-well plate, and incubated for 24 h after which the number of cells that had adhesion capacity was determined [63,64]. On the other hand, the attached remnant cells were collected with 0.25% trypsin–EDTA at 37 °C, stained with trypan blue, and counted. The percentage of adhesion and dead cells was calculated for each treatment condition.

### 2.15. Cell Viability Assay/Cytotoxicity of A. carambola Extract in Combination with Doxorubicin

For the cell viability assay evaluating the cytotoxicity of *A. carambola* ethanolic extract in combination with doxorubicin, MDA-MB-231 cells were seeded at an initial density of 1 × 10^4^ cells/well in a 96-well plate. The cells were treated for 48 h with a combination of 1/5th of IC_50_ of doxorubicin [0.4 μM (0.231 mg/mL)] and concentrations of *A. carambola* ethanolic extract of 15, 25, and 50 μg/mL in medium supplemented at 1% of serum, with a final volume of 100 μL per well. Following treatment, the medium was discarded, and cells were incubated with 1*X* Alamar BlueTM cell viability assay reagent (Sigma-Aldrich, Saint Louis, MO, USA) in a serum-free medium for 4 h. Optical density was measured using a microplate reader (iMark^TM^, Bio-Rad, Hercules, CA, USA) at excitation and emission wavelengths of 570–600 nm. The cytotoxic percentage was calculated as described previously in Section 2.11.

### 2.16. In Silico Analysis

A theoretical analysis was conducted on doxorubicin alone and in combination with *A. carambola* extract. A total of 41 molecular descriptors were calculated using the SwissADME platform to evaluate their physicochemical, pharmacokinetic, and drug-likeness properties [65]. The physicochemical parameters included molecular weight (MW, g/mol), the number of heavy atoms, the fraction of sp^3^ carbons, the number of rotatable bonds, hydrogen bond acceptors and donors, molar refractivity (MR, cm^3^/mol), and topological polar surface area (TPSA, Å^2^). For lipophilicity, descriptors such as iLOGP, XLOGP3, WLOGP, MLOGP, Silicos-IT Log P, and the consensus Log P were calculated [66]. Water solubility was analyzed through ESOL Log S, Ali Log S, and Silicos-IT Log S (expressed logarithmically), alongside their respective solubility values in mg/mL and mol/L. The pharmacokinetic properties assessed included gastrointestinal (GI) absorption, blood–brain barrier (BBB) permeability, P-glycoprotein (Pgp) substrate prediction, and cytochrome P450 enzyme inhibition potential (CYP1A2, CYP2C19, CYP2C9, CYP2D6, and CYP3A4). To determine drug likeness, the compounds were evaluated against the Lipinski, Ghose, Veber, Egan, and Muegge criteria, in addition to bioavailability scores, PAINS alerts, Brenk structural alerts, lead-likeness parameters, and synthetic accessibility values [67].

### 2.17. Statistical Analysis

The results are presented as the mean ± standard deviation (SD). For the cell viability assay, the data represent the average of three independent experiments each conducted with seven replicates. Similarly, for the clonogenic, wound healing, and adhesion assays, the results correspond to the average of three independent experiments, each performed with three replicates. Statistical differences between groups were evaluated using the Mann-Whitney U test [68]. All analyses were performed using GraphPad Prism version 8.0.1 for Windows accessed on 29 April, 17 and 21 October 2024, and 6 and 15 March 2025 (GraphPad Software, San Diego, California, USA, www.graphpad.com (accessed on 20 December 2024)).

## 3. Results

### 3.1. The Ethanol Extract of A. carambola Leaves Contains Flavonoids, Saponins, and Steroids, and Exhibits Reducing and Antioxidant Capacities

A yield of 8.42% was obtained from the leaves subjected to corresponding ethanolic maceration. Preliminary phytochemical analysis revealed the presence of secondary metabolites such as flavonoids, saponins, and steroids (Table 1). The spectroscopic analysis was performed employing a small amount of the EE *A. carambola* (ethanolic extract of *A. carambola*). UV scanning (Figure S1) evidenced peaks at 227, 278, 301, and 380 nm, which could be consistent with flavonoid skeletons such as flavanones, flavones, and flavonols [69]. Additionally, prominent peaks detected between 382 and 418 nm may suggest the presence of chalcone-type skeletons, possibly related to compounds similar to carambolasides, which are abundant in the leaves of this species [36].

The obtained IR spectrum from the FTIR technique is presented in Figure S2. In this spectrum, the following signals were observed at 3279 cm⁻^1^, which could correspond to the stretching of O–H groups, while the signals at 2850, 2927, and 2967 cm⁻^1^ may be associated with C–H stretching from alkyl groups. The peak at 1718 cm⁻^1^ could suggest the presence of a carbonyl group (C=O), and the signal at 1603 cm⁻^1^ might correspond to C=C stretching in aromatic rings. The signals at 1443 and 1371 cm⁻^1^ may indicate C–H bending or aromatic ring vibrations, while those at 1284, 1245, and 1038 cm⁻^1^ could be attributed to C–O stretching, often found in flavonoid structures. Together, these signals may suggest the presence of highly hydroxylated compounds, with some potentially corresponding to chalcone-type flavonoid skeletons [70], aligning with the patterns observed in the UV spectrum, though further confirmation is needed (Figures S1 and S2).

The NMR analysis (Figures S3–S6) provided strong indications of the presence of chalcone-type structures in the extract, although further experimentation is required for definitive identification. In the ^1^H NMR spectrum (Figure S3), signals at δ 7.84, 7.50, 7.41, 7.36, 7.07, 6.92, 6.76, 6.18, and 5.39 ppm correspond to aromatic protons and olefinic signals, which are characteristic of chalcone skeletons. Additionally, the presence of signals at δ 4.92–3.00 ppm suggests possible glycosylation, supporting the idea of a glycosylated chalcone.

The ^13^C NMR spectrum (Figure S4) reinforces this hypothesis with a signal at δ 184.59 ppm, likely representing a carbonyl carbon of the α, β-unsaturated carbonyl system typical of chalcones. The signals between δ 157.86 and 127.96 ppm are consistent with aromatic carbons in conjugated systems, while those at δ 104.14 and 98.04 ppm align with sugar carbons, indicating possible glycosylation. DEPT-90 and DEPT-135 experiments (Figures S5 and S6, respectively) were performed to further support these findings. The DEPT-90 experiment showed signals at δ 130.35, 128.34, 125.18, 116.49, 105.65, 82.97, 77.50, 74.72, 73.79, and 70.29 ppm, reinforcing the presence of aromatic and sugar-like carbons. The DEPT-135 spectrum provided additional evidence with signals at δ 130.38, 128.34, 125.23, 116.50, 92.21, 82.98, 74.74, and 70.48 ppm, which are consistent with the carbon types expected in both chalcone and glycosylated structures. Although these findings strongly suggest the presence of chalcones and their glycosylated derivatives, further experiments, such as HMBC, HSQC, and additional glycosylation-specific tests, are necessary to confirm the exact structures of these compounds in the extract [36,71,72]. This suggests that the ethanolic extract of leaves is rich in flavonoids, which are present in a mixture of carambolaside-like compounds.

The quantification of total flavonoid content (TFC) in the extract revealed a significant presence of 103.17 ± 7.84 μg/mg of quercetin equivalents. This finding underscores the extract’s rich flavonoid composition, aligning with preliminary phytochemical analyses and UV scanning, which indicated that these compounds are the predominant constituents. Furthermore, the antioxidant capacity and iron-reducing ability of the extract were measured at 3.69 ± 0.37%. These activities may be attributed to the presence of glycosylated compounds, suggesting a complex interplay of phytochemicals. The total antioxidant capacity (TAC) was recorded at 45.18 ± 1.27% and total phenolic content (TPC), calculated as gallic acid equivalents (GAE), reached 667.00 ± 11.00 μg/mg. Notably, the DPPH scavenging effect was found to be 50.59 ± 5.28%, indicating robust antioxidant activity. The presence of a purple precipitate in the Marini Bettolo test further suggests the extraction of specific reactive compounds of chalcone type [73].

### 3.2. Chromatographic Analysis

**Figure 1:**
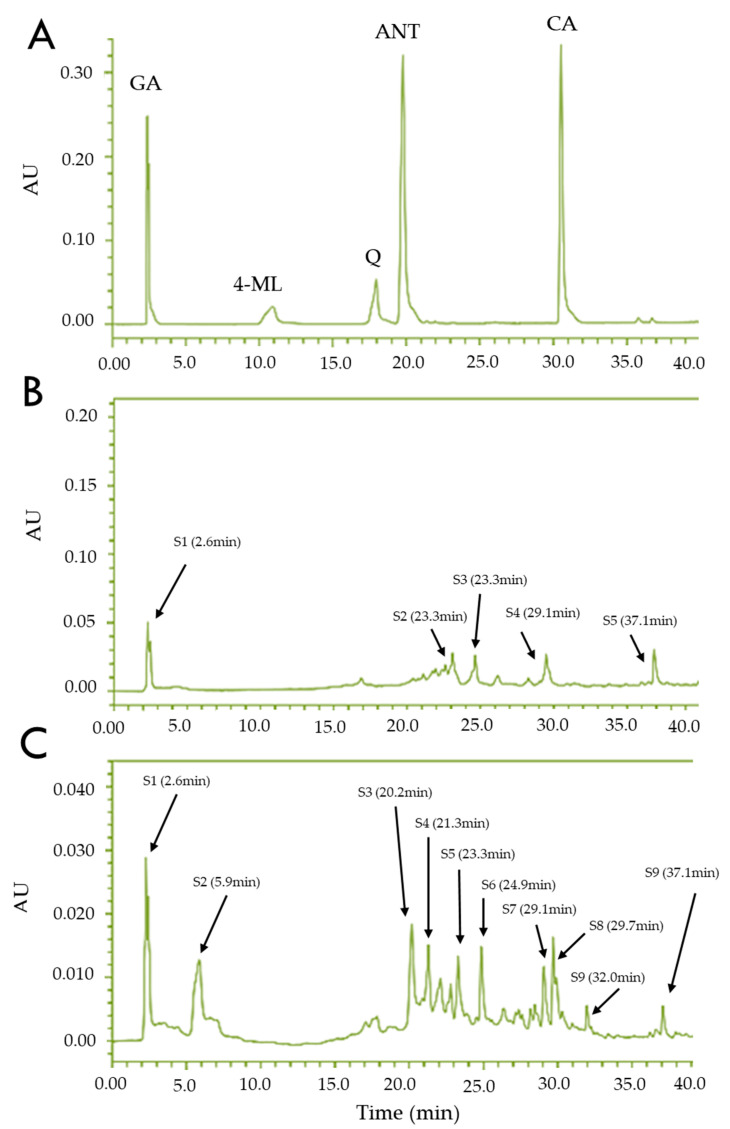
Chromatograms obtained at 290 nm from HPLC analysis. (**A**) Chromatogram of standards: gallic acid (GA, Rt 2.385 min), cinnamic acid (CA, Rt 30.795 min), anthrone (ANT, Rt 20.000 min), quercetin (Q, Rt 17.955 min), and 4-methylumbelliferone (4-ML, Rt 10.908 min). (**B**) Chromatogram of the ethanolic extract of *A. carambola* (500 ppm). (**C**) Chromatogram of the hydrolysate of the leaves of *A. carambola* (500 ppm). S: signal.

Figure 1 shows three HPLC chromatograms analyzed at 290 nm, highlighting the chemical composition of *A. carambola* ethanolic extract and the impact of acid hydrolysis. This chromatogram represents the reference standards gallic acid (GA), 4-methylumbelliferone (4-ML), quercetin (Q), anthrone (ANT), and cinnamic acid (CA), with distinct retention times (Figure 1A). These standards serve as benchmarks for identifying related compounds in the extract. This chromatogram depicts the ethanolic extract of *A. carambola* at 500 ppm (Figure 1B). Peaks corresponding to various compounds, including some similar to quercetin and cinnamic acid, highlight the plant’s complex chemical composition and polyphenolic content. Acid hydrolysis was performed following the methodology proposed by Ross et al. (2009) [57]. Briefly, 0.2 g of extract was hydrolyzed with 2.5 mL concentrated HCl (12 N) containing 1% ascorbic acid and 10 mM EDTA, with 5 mL distilled water added prior to acidification. The mixture was incubated at 85 °C for 30 min, and liberated phenolic acids were extracted with 15 mL chloroform (1:1, *v*/*v*). After vortexing and centrifugation at 7000× *g* for 10 min, the organic layer was collected, evaporated under rotary vacuum, and re-dissolved in 1.5 mL acetonitrile before dilution to 5 mL.

The chromatographic analysis of the hydrolyzed extract (Figure 1C) reveals significant changes in the chemical composition of the ethanolic extract after acid hydrolysis. This treatment, based on the methodology proposed by Ross et al. (2009) [57], involved breaking glycosidic bonds, releasing aglycones, and altering the chromatographic profile. The retention times and UV absorption maxima provide critical evidence for the presence of both shared and unique compounds in the hydrolysate compared to the original extract. One of the shared compounds is evident in Signal 3, with a retention time of 20.2 min and UV absorption maxima at 205.0, 273.5, and 345.1 nm. The absorption at ~345 nm suggests the presence of compounds with extended conjugation, such as chalcones or flavonols. This alignment indicates that the compound persisted after hydrolysis, likely reflecting its stable aglycone form. Similarly, Signal 6 at a retention time of 24.9 min, with UV maxima at 278.3 nm, appears in both the original extract and the hydrolysate, hinting at another flavonoid or polyphenolic compound resistant to hydrolysis. Additionally, Signal 8 at 29.7 min, with a UV maximum at 290.2 nm, is present in both profiles, suggesting another common structural element, possibly a polyphenol with moderate conjugation. The hydrolysate also displays unique signals not observed in the ethanolic extract, such as Signal 4 (21.3 min, UV max at 315.2 nm), which may correspond to a hydrolyzed derivative or aglycone fragment formed during the process. Similarly, Signal 2 at 5.9 min (UV maxima at 271.5, 294.2, and 363.9 nm) exhibits absorption features indicative of highly conjugated structures, possibly representing newly liberated phenolic acids or flavonoid derivatives. The absorption patterns of several signals align with chalcone-type structures, known for their characteristic UV maxima around 340–370 nm. Signals like Signal 3 (345.1 nm) and Signal 2 (363.9 nm) strongly suggest the presence of such compounds in the hydrolysate. These results indicate that hydrolysis not only liberated aglycones but also facilitated the detection of potential chalcone derivatives that were initially glycosylated or bound in complex forms.

Considering the analyses performed, including HPLC and hydrolysis data, along with the specific points identified—such as the presence of functional groups (O–H, C=O, and aromatic C=C), characteristic UV–Vis absorption peaks (227–418 nm), and structural features of flavonoids and chalcones observed in NMR—we could hypothesize that the extract contains bioactive compounds with antioxidant potential. However, further experiments are required to confirm these findings and fully characterize the compounds.

### 3.3. Ethanolic Extract of A. carambola Leaves Affects Specifically the Viability of Breast Cancer Cell Line MDA-MB-231

**Figure 2:**
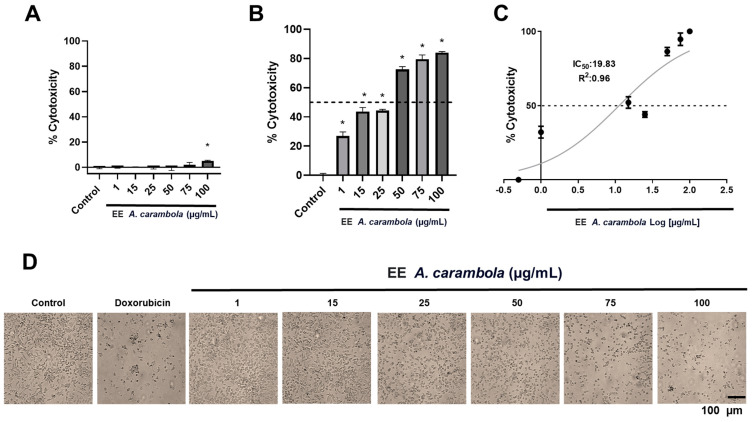
Viability experiments employing ethanolic extract of *A. carambola* on MDA-MB-231 cells. (**A**) No changes in viability were observed in cervical cancer cell line TC-1 exposed to A. carambola extract in increasing concentrations. (**B**) A concentration-dependent effect was observed on MDA-MB-231 cell line exposed to the extract. (**C**) The ethanolic extract of *A. carambola* leaves had an experimental IC_50_ of 20.83 μg/mL in triple-negative breast cancer cell line. (**D**) Morphological changes and detached cells were observed from the concentration of 25 μg/mL of ethanolic extract. Magnification 10×. The *p*-values correspond to significant differences compared to the control, DMEM-F12 medium with 0.1% DMSO, * *p* < 0.05.

The effect of the extract on the cell viability was tested on two cell lines, the TC-1, extensively used as a model for studying cervical cancer due to its expression of HPV16 E6 and E7 oncogenes, and the MDA-MB-231 cell line, representative of the mesenchymal stem-like subtype of TNBC [74,75]. In the TC-1 cell line, no substantial changes in viability were observed at the concentrations tested, with a maximum cytotoxicity effect of 4.26 ± 0.59% at the highest concentration of 100 μg/mL (Figure 2A).

Instead, in the MDA-MB-231 cell line, a concentration-dependent effect was observed. The extract reduced cell viability from the lower concentrations tested, at 1 μg/mL by 26.9 ± 3.3% at 15 μg/mL by 43.7 ± 3.3%, while the 50 μg/mL concentration induced a 72.5 ± 2.3% reduction, the 75 μg/mL of 79.5 ± 3.5%, and 100 μg/mL of 84.0 ± 0.98% (Figure 2B). All concentrations showed a statistically significant difference compared to the control with a *p*-value of 0.0286 (Figure 2B). The maximum inhibitory concentration for the ethanolic extract of A. carambola was calculated at 20.83 ± 1.3 μg/mL (R2 = 0.96; 95% IC = 12.29 to 29.61) (Figure 2C). Furthermore, cells were observed to change from a fibroblast-like morphology to a less elongated shape and even to round cells, without prolongations, from the concentration of 25 μg/mL (Figure 2D). After 48 h of exposure, cells treated with the highest concentration of A. carambola showed morphological changes and cell density similar to cells treated with 2 μM doxorubicin, with a few adherent cells and a round morphology (Figure 2D). This suggests that the observed activity is type-specific, so that there may be mechanisms of action of the metabolites present in the leaf extract that are particular to TNBC cells.

### 3.4. The Ethanolic Extract of A. carambola Leaves Decreases the Clonogenic Survival of MDA-MB-231 Cells

**Figure 3:**
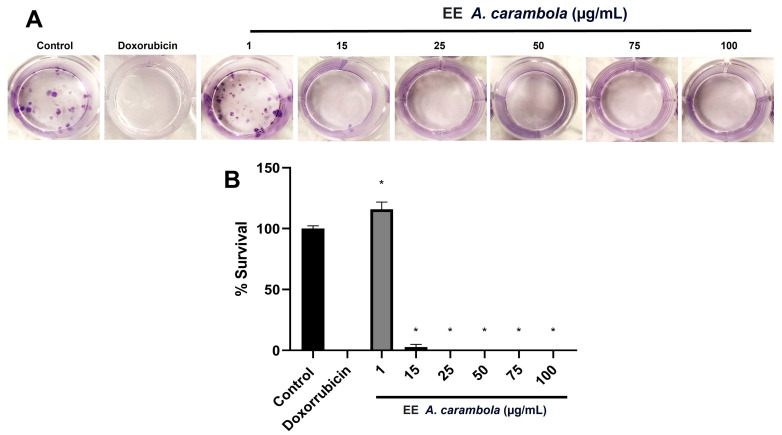
Ethanolic extract of *A. carambola* leaves decreases replicative immortality of MDA-MB-231 cells. (**A**) Photographs depict the number of colonies formed after the exposition of each treatment. It is observed that a concentration-dependent effect completely inhibits cell survival. (**B**) The graph shows the percentage of survival treatment. The *p*-values correspond to significant differences compared to the control, only DMEM medium, * *p* < 0.05.

The ability of the extract to interfere with the replicative immortality/proliferation of MDA-MB-231 cells was analyzed by clonogenic assay. It was observed that colony formation decreased in a concentration-dependent manner (Figure 3A). The 15 μg/mL concentration reduced survival to only 2.63 ± 2.27%, and this was statistically significant compared to the control, *p* = 0.0286. This reduction persisted until complete inhibition at higher concentrations (Figure 3B). It should be noted that this effect was similar to that of doxorubicin chemotherapy, which also completely inhibited colony formation compared to the control (*p* = 0.0286). This suggests that metabolites present in *A. carambola* leaf extract may be regulating cellular processes leading to inhibition of replicative immortality/proliferation of MDA-MB-231 cells.

### 3.5. A. carambola Extract Interferes with MDA-MB-231 Cells the Migration

**Figure 4:**
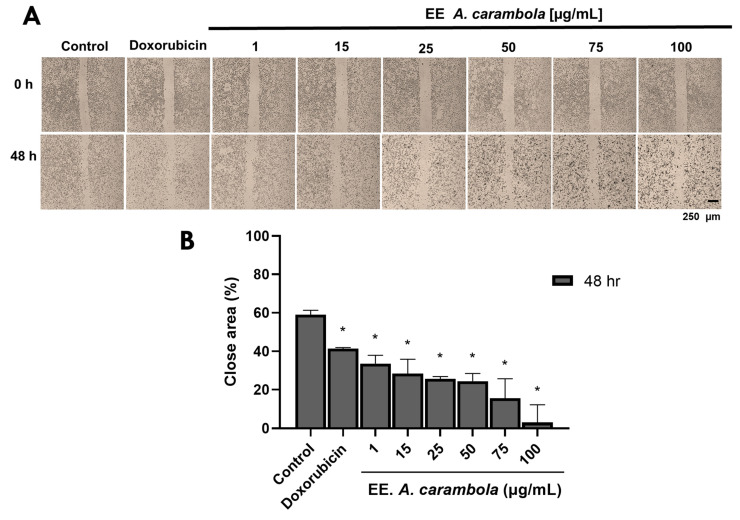
Ethanolic extract of *A. carambola* leaves interferes with MDA-MB-231 cell migration. (**A**) Images captured at 48 h of the wound area made in MDA-MB-231 cell monolayers. Magnification 4×. (**B**) The graph shows the changes in the open area; a concentration-dependent inhibitory effect can be observed at 48 h that was superior to the doxorubicin effect. Comparison to 48 h control, * *p* < 0.05.

The ethanolic extract of *A. carambola* induced changes in the migratory capacity of MDA-MB-231 cells (Figure 4A,B). A well-defined concentration-dependent effect was observed at 48 h. Compared to the control and doxorubicin, in which the area of closure was increased by up to 58.92 ± 2.34% and 41.37 ± 0.59%, respectively, the extract to 1 μg/mL had a value of 33.59 ± 4.39%, at 15 μg/mL of 28.49 ± 7.37%, at 25 μg/mL of 25.72 ± 1.17%, at 50 μg/mL of 24.44 ± 4.04%, at 75 μg/mL of 15.50 ± 10.23% and at 100 μg/mL of 3.06 ± 9.17% (Figure 4B). That is, in the monolayer treated with the extract, the area of closure remained below the values of the area of closure of the controls, and these were, in all cases, statistically different, with a *p*-value of 0.0286. This suggests that *A. carambola* leaf extract has an inhibitory effect on the migration of these TNBC cells.

### 3.6. A. carambola Ethanolic Extract Affects Cell Adhesion: MDA-MB-231 Adhesion Was Affected in a Concentration-Dependent Manner

**Figure 5:**
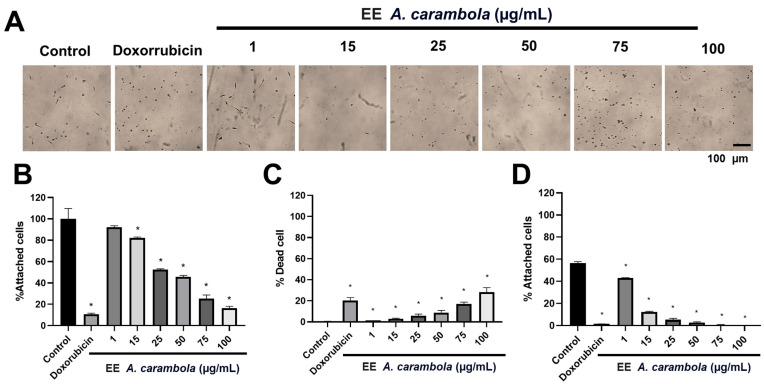
The ethanolic extract of *A. carambola* leaves affects the cell adhesion of MDA-MB-231 cells. (**A**) The micrographs show the adhesive capacity of cells recovered after exposure to *A. carambola* extract and reseeded for 24 h. The adhesive capacity decreases as the concentration of the extract increases. Magnification 10×. (**B**) The graphs show the percentage of cells adhered to the monolayer after being treated with the extract for 48, showing a concentration-dependent decrease in adhesion. (**C**) The graph shows the percentage of cell death after 48 h of treatment. (**D**) The graph shows the percentage of adhesion of detached cells after treatment that were recovered and reseeded. The *p*-values correspond to significant changes compared to the control, * *p* < 0.05.

Firstly, we observed the detachment of the cells from the monolayer during exposure to *A. carambola* leaf extract. From the concentration of 25 μg/mL, the number of unattached cells was significantly different from the control, with 52.59 ± 0.64% of cells remaining adhered, with a *p*-value of 0.0286 (Figure 5A, B). At 50 μg/mL of extract, only 45.67 ± 1.35% was in the monolayer, at 75 μg/mL 25.32 ± 3.43%, and at 100 μg/mL, only 16.23 ± 1.94% remained attached to the cells (*p* = 0.0286, for all the conditions). Compared to doxorubicin, the different concentrations of the extract did not have a greater effect than that generated by the chemotherapeutic, in which monolayers exposed to it only 10.71 ± 0.97% of cells remained adherent (Figure 5A,B). We were interested in analyzing the viability of the detached cells by using the supravital dye trypan blue to determine whether the detachment corresponds to dead or living cells that lose their adhesion capacity. Interestingly, we observed that the percentage of dead cells was relatively low compared to the detachment percentage. For concentrations of 25, 50, 75, and 100 μg/mL, the dead cells’ percentages were 5.69 ± 1.71%, 8.83 ± 2.12%, 17.18 ± 1.59%, and 28.26 ± 4.27%, respectively, suggesting a direct effect on the binding of cells to the substrate and to each other, not due to cell death per se. This effect is comparable to that of doxorubicin, where the number of death cells was also not high, 20.35 ± 2.65%.

To test for direct damage to cell adhesion mechanisms, we subsequently analyzed whether these detached and viable cells could re-attach to the treated surface of the culture dish [46]. The results showed that the cells had very low percentages of adhesion compared to the number of live cells (Figure 5C,D). At 25 μg/mL, adhesion was 5.26 ± 1.38%, at 50 μg/mL, it was 2.76 ± 0.562%, at 75 μg/mL, it was 0.36 ± 0.62%, and at 100 μg/mL, no attached cells were observed (Figure 5D). This was statistically different from the control for all concentrations (*p* = 0.0286). It should be noted that 75 and 100 μg/mL concentrations had even lower adhesion values than doxorubicin, which had an adhesion percentage of 1.49 ± 0.04%, and that this was statistically significant with a *p*-value of 0.0286 for both cases. These results indicate that there is possibly a direct inhibitory effect on mechanisms that allow cell adhesion.

### 3.7. The Combination of a Low Dose of Doxorubicin and Intermediate Doses of A. carambola Extract Reduces MDA-MB-231 Cell Viability

**Figure 6:**
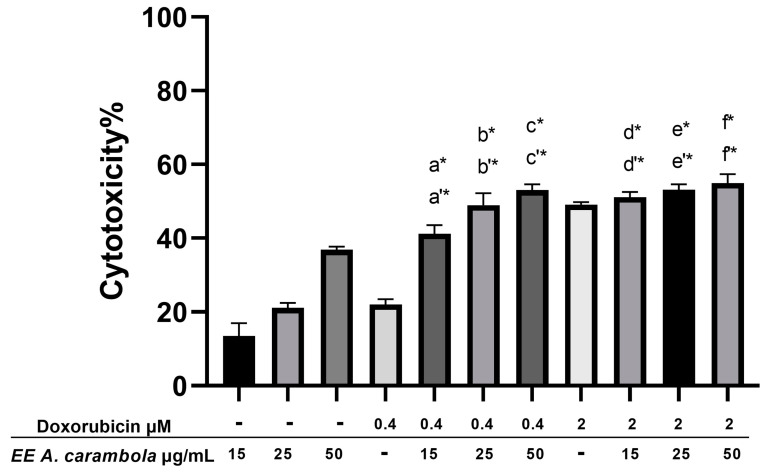
The combination of a low dose of doxorubicin and intermediate doses of *A. carambola* extract reduces the cell viability of MDA-MB-231 cells. The graph shows the reduction in cell viability induced by the different combinations after 48 h of treatment. An additive effect was observed between the 1/5 IC_50_ dose of doxorubicin (DOX) and the three tested concentrations of the extract. ^a^ 0.4 μM DOX + 15 μg/mL extract vs. 15 μg/mL of the extract, ^b^ 0.4 μM DOX + 25 μg/mL vs. 25 μg/mL, ^c^ 0.4 μM DOX + 50 μg/mL vs. 50 μg/mL, ^d^ 2 μM DOX + 15 μg/mL vs. 15 μg/mL, ^e^ 2 μM DOX + 25 μg/mL vs. 25 μg/mL, ^f^ 2 μM DOX + 50 μg/mL vs. 50 μg/Ml, ^a’^ 0.4 μM DOX + 15 μg/mL vs. 0.4 μM DOX, ^b’^ 0.4 μM DOX + 25 μg/mL vs. 0.4 μM DOX ^c’^ 0.4 μM DOX + 50 μg/mL vs. 0.4 μM DOX, ^d’^ 2 μM DOX + 15 μg/mL vs. 2 μM DOX, ^e’^ 2 μM DOX + 25 μg/mL vs. 2 μM DOX, ^f’^ 2 μM DOX + 50 μg/mL vs. 2 μM DOX, * *p* < 0.05.

To elucidate whether *A. carambola* leaf extract could be an adjuvant therapy to help reduce doses of doxorubicin in the treatment of TNBC, we tested whether a low dose of the chemotherapeutic in combination with extract concentrations impacts the viability of MDA-MB-231 cells (Figure 6). One-fifth of the IC_50_ of doxorubicin, equivalent to 0.4 μM, was used, and the concentrations of 15, 25, and 50 μg/mL of *A. carambola* leaf extract. These were chosen because they are the concentrations at which the inhibitory effects on hallmarks of cancer of MDA-MB-231 cells began to be observed.

One-fifth of the IC_50_ of doxorubicin decreased cell viability slightly with a cytotoxicity percentage of 22.01 ± 1.76%. However, when the cells were treated with the combination of this and the extract, the decrease in cell viability was consistently increased and statistically significant in all the cases (*p* = 0.028). In conjunction with the *A. carambola* leaf extract at the concentration of 15 μg/mL the cytotoxicity percentage augmented to 41.17 ± 2.94%. This result suggests an additive effect, as the percentage of cytotoxicity for this concentration of extract alone was 13.45 ± 4.28%. A similar result was observed with the 25 μg/mL concentration, which in combination with doxorubicin increased the percentage to 48.98 ± 3.90%, while it only had a value of 21.13 ± 1.64%. It should be noted that the combination with the concentration of 50 μg/mL had higher values than the IC_50_ dose of doxorubicin, which was 49.08 ± 0.90% (Figure 6). The extract alone had a cytotoxicity percentage of 36.86 ± 1.02%, whereas with the chemotherapeutic, this increased to 53.00 ± 2.00%, and this was statistically significant in comparison with doxorubicin IC_50_, with a *p*-value of 0.0286. These results lead us to believe that *A. carambola* leaf extract could indeed be an adjuvant therapy to reduce the dose of chemotherapy, with all that this implies in terms of side effects.

In the case of combinations of the extract with the IC_50_ of doxorubicin (2 µM), this effect was not observed on the same scale. There was a slight increase in the percentage of cytotoxicity, which although statistically different from the value induced by the IC_50_ dose of the chemotherapeutic alone, of 49.08 ± 0.90%, did not correspond to the sum of the two effects. For the combination with 15 μg/mL of the extract, the percentage was 51.08 ± 1.81%, with 25 μg/mL of 53.08 ± 1.91%, and with 50 μg/mL was of 54.98 ± 2.88% (Figure 6). This suggests that there exists a dose range in which there is an additive effect, but when this is exceeded, it is canceled out.

The physicochemical parameters of doxorubicin were obtained using SwissADME, a web-based tool for predicting molecular properties and pharmacokinetics. A total of six main parameters were evaluated under standard input conditions, including the chemical structure of doxorubicin, to determine its lipophilicity (Log P), polar surface area (TPSA), solubility, and interaction with molecular transporters. These parameters were analyzed both individually and in combination with an extract of *A. carambola* to understand potential synergistic effects on the compound’s absorption and intracellular accumulation. The physicochemical parameters of doxorubicin, such as its high polarity reflected in a TPSA of 185.84 Å^2^, moderate lipophilicity (Log P close to 2.85), and its characteristic as a substrate of P-glycoprotein (P-gp), support its limited ability to cross cellular membranes and accumulate inside cells. This limited permeability and intracellular accumulation may determine its therapeutic efficacy, as it hinders doxorubicin from reaching optimal concentrations within the cell to exert its cytotoxic effect [76,77].

Under the hypothesis that some components of the *A. carambola* extract are polyphenols, it is suggested that these molecules might be modulating the intracellular environment to favor the absorption and accumulation of doxorubicin. Polyphenols are well documented as antioxidants and modulators of cellular transport proteins, particularly transporters like P-gp, which is responsible for expelling doxorubicin out of the cell. By reducing the activity or expression of P-gp, polyphenols could improve the intracellular retention of doxorubicin, allowing its cytotoxic effect to be comparable to that observed with higher concentrations, such as 2 µM [78]. In this context, the physicochemical parameters most affected include the polarity and low lipophilicity of doxorubicin, which limit its passive permeability across the cell membrane. At the same time, being a P-gp substrate exacerbates this limitation, as it facilitates the active expulsion of the drug. If polyphenols from the *A. carambola* extract act by modulating these transporters or improving membrane fluidity, they could create more favorable conditions for doxorubicin absorption, optimizing its intracellular bioavailability and, consequently, its cytotoxic effect. Therefore, the observed synergistic effect in the combination of doxorubicin with the extract at different concentrations may be explained by this improvement in the intracellular accumulation of the drug, allowing for cytotoxic levels similar to those observed with higher doses of doxorubicin alone.

## 4. Discussion

This study determined that the ethanolic extract of *A. carambola* leaves had inhibitory effects on some of the hallmarks of triple-negative breast cancer cell line MDA-MB-231. In a concentration-dependent manner, the extract induced changes in the morphology of these cells, shortened cell prolongations, and reduced viability, clonogenic survival, cell migration, and adhesion. To the best of our knowledge, there are no previous studies that have evaluated the activity of this type of extract on MDA-MB-231 cells. However, some antecedents show that other parts of the plant have selective anticarcinogenic activity. Regarding cell viability and anti-proliferative activity, it has been reported that methanolic extract leaves induced apoptosis in Ehrlich’s ascites carcinoma cells through the p53/BAX/Bcl2 signaling pathway, thus reducing cell viability, and that roots of hydroalcoholic extract can arrest cell cycle in the G1 phase in human breast carcinoma cell lines MCF-7, BT-20, and MDA-MB-231, and not in normal cells such as the lines HMEC, MCF-10A, HPL1A, and HUVEC [30,79]. Specifically, it has been characterized that the compound 2-Dodecyl-6-methoxycyclohexa-2,5-diene-1,4-dione (DMDD), isolated from the roots affects the colony formation of the murine breast cancer cell line 4T1, through the regulation of the MAPK signaling pathway [47]. While in breast cancer cells MDA-MB-231, MCF-7, and BT-20, and lung carcinoma and osteosarcoma cell lines, it was demonstrated that DMDD induces sustained ROS overproduction leads to inhibition of the cell cycle transition from G1 to the S phase, apoptosis, and canonical NF-κB pathway inhibition [30].

Our results are consistent with these experimental findings, so the same regulation may occur. Therefore, it will be interesting to analyze changes in ROS production, the cell cycle, the MAPK, and the apoptotic pathways, and in the NF-κB pathway. Regarding the impact of *A. carambola* leaf extract on migration, we characterized a concentration-dependent effect. This suggests that there may be a direct effect on the signaling pathways that regulate this biological process. In this regard, it has been reported that DMDD inhibited this hallmark of cancer and invasion in murine breast cancer cells 4T1 at 24 h, and that this is mediated by the MAPK signaling pathway and decreased expression of metalloproteases 2 and 9 [30]. Therefore, as subsequent steps in our research, it must be established whether these mechanisms are responsible for the effects generated by the leaf extract or whether they differ from the mechanism of action of DDMD.

On the other hand, while we did not characterize the effect of the extract on invasion, we did establish its impact on adhesion. We were interested in analyzing this hallmark of cancer because it is known that cancer cells have a stronger adhesion strength to the ECM than normal cells and because during viability assays, we observed the detachment of MDA-MB-231 cells from the monolayer. The results showed that cells treated with the extract detached from the monolayer without significant cell death but lost their ability to reattach to a substrate in direct proportion to the increase in extract concentration. This suggests that the extract may be regulating the expression of adhesion and extracellular matrix-binding proteins, such as integrins, cadherins, and selectins [80]. This finding is important because altering the adhesion capacity of cancer cells can impact their proliferation and their ability to establish metastatic tumors.

In summary, the results of this work show that ethanolic extract of *A. carambola* leaves inhibits the viability, survival/proliferation, migration, and adhesion of MDA-MB-231 cells representative of the mesenchymal stem-like subtype of TNBC. This undoubtedly opens the way for further research to establish the composition of the extract. It becomes mandatory to identify the bioactive components and determine their biological activity separately and together. It is also necessary to establish their mechanism of action and their safety as a first step in pre-clinical research. In addition, given previous reports on the biological activity of compound DDMD, it also becomes necessary to determine if is present in the leaf extract, in what quantity, and whether it is the metabolite responsible for the activity or a combination of several active metabolites.

In this regard, preliminary phytochemical screening and ^1^H NMR analysis performed in this study suggested the presence of flavonoids, saponins, and steroids in the extract, while NMR signals the presence of carambolasides. The presence of chalcone-type flavonoids was also supported by typical UV absorption maxima for these skeletons [27,37,71]. In the evaluation of the antioxidant and biological activities of the ethanolic extract of leaves, it is important to consider that while flavonoids, particularly glycosylated compounds like carambolasides, may play a significant role, other phytochemicals could also contribute to the observed effects. Studies have reported the presence of various quercetin derivatives, catechins, and their glycosides in the leaves and stems of the same species. For instance, research by Wu et al. (2022) identified several quercetin glycosides in extracts from the leaves, which demonstrated notable antioxidant and anti-inflammatory properties [81]. Similarly, catechins have been linked to various health benefits, including enhanced antioxidant activity and protective effects against oxidative stress, as noted in studies by Sheng et al. (2023) [82]. These compounds, alongside carambolasides, could synergistically contribute to the extract’s overall efficacy. Their presence suggests that the biological activities may not solely be due to flavonoids but could also stem from the combined effects of multiple constituents within the extract [79]. The interaction between these different classes of phytochemicals could enhance the extract antioxidant capacity and provide additional protective effects. Given this complexity, it is crucial to conduct further studies that focus on the identification and isolation of these individual flavonoids, catechins, and their derivatives. Understanding the roles and mechanisms of action of these compounds could offer a more comprehensive view of the extract’s therapeutic potential and allow for the development of targeted applications in herbal medicine. This multifaceted approach will enrich our understanding of how various phytochemicals contribute to the antioxidant and biological activities observed in the extract, highlighting the potential of the plant as a source of beneficial compounds for health promotion.

In the results, a comparison was made between the chromatograms of *A. carambola* extract before and after acid hydrolysis, highlighting the release of aglycones and the appearance of compounds not observed in the total ethanolic extract after this treatment. These results provide evidence that acid hydrolysis not only released stable aglycones but also facilitated the detection of possible chalcone derivatives that were previously glycosylated or in complex forms [57]. It was observed that peaks corresponding to compounds, likely flavonoids based on their UV absorption maxima, as well as phenolic compounds, persisted or allowed detection in their free form after the treatment, emphasizing their presence in the extract [42,83,84,85]. This suggests that these compounds are indeed present, and the biological effects attributed to the ethanolic extract of *A. carambola* may be linked to them. It would be interesting to further investigate this extract in an in vivo system, where the high complexity of the system, coupled with its enzymatic and non-enzymatic systems [86], also the gut microbiome [87], could help release these compounds, allowing them to exert a biological effect. Another point to highlight is the use of the solvent employed; ethanol was chosen as the solvent for preparing the extract due to its widespread availability in communities and its common use in ethnomedical practices [88]. While other solvents like methanol, chloroform, or hexane could be used to extract different compounds, ethanol is safer and more reflective of traditional usage, making it the most suitable choice for this study [27].

An interesting point that this study also focuses on is the potential of the extract as an adjuvant in the treatment of triple-negative breast cancer (TNBC). To probe this hypothesis, we analyzed the impact of combining the extract with a dose of doxorubicin equal to one-fifth of the IC_50_ dose. We found an additive effect between the 15, 25, and 50 µg/mL concentrations and the low dose of doxorubicin, which achieved the same viability decrease values as the IC_50_ of the chemotherapeutic alone. However, this did not occur when the same concentrations were combined with the complete IC_50_ doses, suggesting, as noted above, that there may be a dose range in which there is an additive effect, but when this is exceeded, it is canceled out. It is therefore important to consider analyzing this effect in depth to determine the active range in which the extract, or its components, can be an adjuvant treatment. To our knowledge, this is the first study where this possible application of *A. carambola* is considered, and this is relevant because it may imply a decrease in the clinical doses of chemotherapeutics and in their side effects or resistance and all that this means for the comfort of the patients. Of course, it becomes necessary to evaluate these combinations in in vivo models to understand the behavior and efficacy of treatments more deeply in a more complex and realistic biological environment. In vivo studies could provide additional information on the bioavailability, pharmacokinetics, and potential systemic effects of combining the extract with doxorubicin, offering a more comprehensive perspective on its potential clinical use for this type of cancer that has limited therapeutic options. It is worth noting here that Saghir and Cols. reported that methanolic extract of the leaves at 5000 mg/kg administered orally to Sprague Dawley rats was safe in an acute toxicological assay, and in 250, 500, and 1000 mg/kg concentrations in the sub-chronic toxicological analysis [89]. Therefore, similar results could be expected with the ethanolic extract.

In examining the chemical aspects of the study on the ethanolic extract of leaves, several limitations must be acknowledged. One significant limitation is the variability of secondary metabolites, which can change depending on the geographical area and the season. This variability can influence the composition and biological activity of the extract, highlighting the need for future studies aimed at understanding the metabolomics of these compounds throughout different growth stages and environmental conditions. Another limitation is the extraction method used in the study. While an ethanolic extract provides valuable insights into the phytochemical profile, replicating the effects in an aqueous extract—similar to traditional preparations used by local populations—could serve as a useful comparison [26,90]. This approach may help validate the observed activities and enhance the understanding of how different extraction methods influence bioactivity. Additionally, even if a similar profile of phytochemicals is obtained, it is essential to conduct bioassays and isolate the primary metabolites responsible for the observed effects. This isolation process would enable researchers to pinpoint which specific compounds contribute most significantly to the extract’s biological activity, facilitating a more thorough understanding of its mechanisms. Furthermore, establishing studies to elucidate the structure of the predominant metabolite is crucial. Understanding its chemical structure and how it interacts with other compounds, such as doxorubicin, could provide insights into potential synergistic effects or antagonistic interactions that might enhance therapeutic efficacy. Finally, incorporating in vivo studies would be valuable in confirming the biological effects observed in vitro, offering a more comprehensive view of the extract’s potential as a therapeutic agent.

Although the results of this work clearly show the anticancer potential of *A. carambola* leaf extract in the in vitro evaluation, some limitations should be mentioned. For example, we did not perform the study considering the counterpart of a non-malignant breast tissue cell line. However, it is worth noting that previous studies report a specific anticancer effect against MCF7, BT2, and MDA-MB-231 breast cancer cells and not on the human mammary epithelial cell lines HMEC and MCF-10A [30]. We also did not explore its effect on other cell lines of other TNBC subtypes, to establish whether there is no distinction between them and therefore whether it could be applied in general in this type of cancer. A further limitation is that we did not analyze other cancer hallmarks usually included in such in vitro studies, namely invasion, by trans-well assays, and angiogenesis, using an endothelial cell tubule formation assay, and could also consider verifying effects in 3D culture. We also did not carry out analyses to try to elucidate any possible mechanism of action of the extract, like the characterization of targets in cellular processes like proliferation/apoptosis, to mention just one example.

In this study, the structural characterization of the main components of the ethanolic extract of *A. carambola* leaves was conducted using NMR, providing qualitative insights into the phytochemical composition. While this approach enabled the identification of possible components through comparative analysis with literature-reported signals, future studies could significantly benefit from the application of high-resolution mass spectrometry (HRMS) [91]. This advanced analytical technique would allow for a more precise and quantitative determination of the main bioactive compounds in the extract, complementing the qualitative data obtained via NMR. The integration of more advanced techniques, like HRMS or GCMS, into the analysis would offer several advantages, including improved sensitivity, the ability to detect minor components, and the potential for more accurate molecular identification through comparison with authentic standards [92]. Notably, recent studies on *A. carambola* extract have employed HRMS and GCMS, enabling comparisons of metabolites and the establishment of more precise metabolomic profiles. This approach could be particularly relevant for evaluating how metabolite composition varies due to geographic differences or environmental stress conditions that may influence the species [93,94]. Furthermore, the use of more advanced techniques could aid in correlating specific bioactive compounds with their observed biological activities, ultimately supporting the development of targeted therapeutic applications.

It is important to mention that although this study was based on a single collection during a specific period, future studies could benefit from exploring variations in secondary metabolites influenced by factors such as climate, soil, and biotic stressors [95]. Establishing a phytochemical profile of *A. carambola* leaves from different regions and conditions using advanced techniques would help identify bioactive compounds, determine those responsible for the biological activity, and develop marker compounds for standardization and monitoring, reinforcing the extract potential as a therapeutic adjuvant.

This study has made significant contributions to the field, demonstrating that the ethanolic extract of *A. carambola* leaves can induce concentration-dependent changes in cell morphology, reduce viability, inhibit clonogenic survival, and prevent migration and adhesion of triple-negative breast cancer cells, MDA-MB-231. It also lays the groundwork for exploring its use as an adjuvant in the treatment of this disease with the chemotherapy drug doxorubicin.

## 5. Conclusions

The ethanolic extract of *A. carambola* leaves, rich in flavonoids, steroids, and saponins, demonstrated a strong antioxidant capacity and a substantial content of flavonoids and phenolics. Additionally, it exhibited a significant reduction in ferric-reducing power and effective DPPH scavenging activity. The EE *A. carambola* reduced the viability of triple-negative breast cancer cell MDA-MB-231, in a concentration-dependent manner, with an IC_50_ of 20.89 μg/mL. In addition, it decreased the survival/replication capacity, migration, and adhesion of these cells. Also, the combination of 15, 25, and 50 μg/mL of the extract with 1/5 IC_50_ doses of doxorubicin had an additive effect to reduce the viability of these cells. This suggests that the phytochemicals present in the extract have biological activity against triple-negative breast cancer, so more exhaustive and specific analyses should be carried out to establish which are the active compounds and the mechanisms of action that modulate these hallmarks of cancer and further exploration of the therapeutic potential of *A. carambola* in in vivo models should be carried out to establish its potential as an adjuvant therapy in the treatment of this cancer with limited therapeutic options.

## Figures and Tables

**Table 1:** Preliminary phytochemical analysis of the ethanolic extract of *A. carambola* leaves.

Metabolites	*A. carambola* Ethanolic Extract
Tannins (FeCl_3_)	−
Tannins (gelatin hydrolysis)	−
Flavonoids (Shinoda test)	+++
Flavonoids (Salkowski test)	+++
Marini Bettolo Test	purple precipitate
Steroids	++
Alkaloids (Dragendorff test)	−
Alkaloids (Wagner test)	−
Alkaloids (Mayer test)	−
Saponins (hemolysis in agar)	+++
Saponins (foam formation)	++
Coumarins (NaOH test)	−
TFC ^a^	QE = 103.17 ± 7.84 µg/mg extract
FRPA ^b^	% age reduction = 3.69 ± 0.37
TAC ^c^	% age TAC = 45.18 ± 1.27
TPC ^d^	GAE = 667.00 ± 11.00 µg/mg extract
DPPH ^e^	Scavenging effect = 50.59 ± 5.28%

+++ appreciable amount (positive within 5 min); ++ = moderate amount (positive after 5 min but within 10 min); − = completely absent. ^a^ TFC (total flavonoid content): expressed in quercetin equivalents (QE) µg/mg of extract. ^b^ FRPA (ferric reducing power assay): expressed as % reducing power relative to the ascorbic acid control. ^c^ TAC (total antioxidant capacity): expressed as % antioxidant capacity relative to the ascorbic acid control. ^d^ TPC: expressed in µg of gallic acid (GAE) for mg of extract. ^e^ DPPH, scavenging effect (%). Concentration at 1.25 mg/mL.

## Data Availability

The datasets used and/or analyzed during the current study are available from the corresponding author upon reasonable request.

## Supplementary Materials

The following supporting information can be downloaded at: https://www.mdpi.com/article/10.3390/pharmaceutics17010002/s1, Table S1. Comparisons of chemical shifts of the AC extract vs. some of the principal metabolites isolated from *Averrhoa carambola* leaves. Figure S1. UV spectra recorded in MeOH at 0.1mg/mL. Figure S2. Infrared spectra (wavenumber in cm^−1^). Figure S3. ^1^H-NMR spectra (400 MHz) of *Averrhoa carambola* extract, recorded in DMSO-d6. Figure S4. ^13^C-NMR spectra (100 MHz) of *Averrhoa carambola* extract recorded in DMSO-d6. Figure S5. DEPT90 experiment of EE of *Averrhoa carambola*, recorded in DMSO-d6. Figure S6. DEPT135 experiment of EE of Averrhoa carambola, recorded in DMSO-d6.
