# Supplementary material for: Ethanolic Extract of Averrhoa carambola Leaf Has an Anticancer Activity on Triple-Negative Breast Cancer Cells: An In Vitro Study
Source: Pharmaceutics. 2024 Dec 24;17(1):2. doi: 10.3390/pharmaceutics17010002 (PMC11768879; doi:10.3390/pharmaceutics17010002)
Supplement: Supplementary file 1 [file pharmaceutics-17-00002-s001.zip › pharmaceutics-3359430-supplementary.pdf]

Table S1. Comparisons of chemical shifts of the AC extract vs some of the principal metabolites isolated from the leaves *Averrhoa carambola* leaves.

	**AC leaves extract**		**Catechin**		**Carambolaside E**	**Carambolaside F**	**Carambolaside G**	**Carambolaside H**	**β-sitosterol**
**Signal**				**Signal**					
H	δ_H	δ_C	δ_H		δ_H	δ_H	δ_H	δ_H	δ_H
1	0.52 s	14.03	2.460	1					0.86-1.10 (5x3H)
2	0.91 s	18.62	2.48	2	7.04 (d, 8.5)	7.05/6.90 (br s)	7.05/6.89 (br s)	7.06/6.92 (br s)	3.53 m
3	1.10-1.14 s	22.18	2.50	3	6.69 (d, 8.5)	6.66 (br s)	6.65 (br s)	6.67 (br s)	5.35 m
4	1.16-1.22 s	25.32	2.52	4					
5	1.41-1.61 s	29.11	2.810	5	6.69 (d, 8.5)	6.66 (br s)	6.65 (br s)	6.67 (br s)	
6	1.81 s	33.78	2.824	6	7.04 (d, 8.5)	7.05/6.90 (br s)	7.05/6.89 (br s)	7.05/6.92 (br s)	
7	1.90 s	38.89	2.825	7	2.85 (2H, dd, 8.4, 7.2)	2.87/2.58 (2H, br s)	2.85/2.65 (2H, br s)	2.86/2.59 (2H, br s)	
8	1.97 s	39.10	3.292	8	3.29 (2H, dd, 8.4, 7.2)	3.27/3.11 (2H, br s)	3.28/3.13 (2H, br s)	3.28/3.11 (2H, br s)	
9	1.68 s	39.31	3.296	9					
10	2.00 s	39.52	3.300	1´					
11	2.05-2.95 s	39.7339.94	3.304	2´					
12	3.02-3.49 s	40.15	3.937	3´					
13	3.60 s	56.19	3.951	4´					
14	3.64 s	60.62	3.957	5´	5.89 (s)	5.83 (br s)	5.85 (br s)	5.83 (br s)	
15	3.73-3.80 s	61.43	3.970	6´					
16	3.96 s	62.23	3.976	1´´	4.85 (d, 9.6)	5.08 (d, 9.2)	5.02 (d, 9.9)	5.07 (d, 9.7)	
17	4.02-4.56 s	63.15	3.990	2´´	4.18 (t, 9.6)	5.83/5.71 (br s)	5.92/5.70 (br s)	5.83/5.70 (br s)	
18	4.72 s	63.46	4.543	3´´	3.68 (dd, 9.6, 2.8)	4.04 (dd, 7.3, 3.0)	3.94 (br d, 9.6)	4.00 (dd, 9.6, 2.8)	
19	4.74 s	69.98	4.526	4´´	3.97 (d, 2.8)	4.03 (d, 3.0)	3.99 (d, 2.9)	4.01 (d, 2.8)	
20	5.17 d	71.75	5.840	5´´	3.77 (q, 6.4)	3.89 (q, 6.3)	3.85 (q, 6.4)	3.85 (q, 6.3)	
21	5.32 s	72.93	5.845	6´´	1.29 (3H, d, 6.4)	1.35 (3H, d, 6.3)	1.32 (3H, d, 6.4)	1.33 (3H, d, 6.3)	

22	5.72 s	74.42	5.913	1´´´	4.53 (d, 7.5)	4.40 (d, 7.6)	4.36 (d, 7.0)	4.39 (d, 7.6)	
23	6.07-6.14 s	77.21	5.919	2´´´	3.56 (dd, 9.7, 7.5)	3.47 (dd, 9.8, 7.6)	3.46 (dd, 9.5, 7.0)	3.48 (dd, 9.7, 7.6)	
24	6.27 s	82.64	6.694	3´´´	3.49 (dd, 9.7, 3.3)	3.39 (dd, 9.8, 3.3)	3.42 (dd, 9.5, 3.1)	3.40 (dd, 9.7, 3.2)	
25	6.53-6.77 s	91.88	6.699	4´´´	3.60 (d, 3.3)	3.57 (d, 3.3)	3.58 (d, 3.1)	3.56 (d, 3.2)	
26	6.87 s	98.04	6.714	5´´´	3.66 (q, 6.4)	3.63 (q, 6.5)	3.61 (q, 6.4)	3.59 (q, 6.3)	
27	7.05 s	102.40	6.719	6´´´	1.27 (3H, d, 6.4)	1.26 (3H, d, 6.5)	1.26 (3H, d, 6.4)	1.25 (3H, d, 6.3)	
28	7.20 s	104.14	6.743	1´´´´					
29	7.40 s	115.14	6.763	2´´´´		7.50 (br s)	7.50/7.37 (br s)	7.36/7.23 (d, 8.5)	
30	7.56 s	127.73	6.824	3´´´´		7.37 (br s)	7.20 (br s)	6.78 (d, 8.5)	
31	7.94 s	127.73	6.828	4´´´´		7.37 (br s)	7.20 (br s)		
32	8.75 s	127.96		5´´´´		7.37 (br s)	7.20 (br)	6.78 (d, 8.5)	
33	9.19 s	129.12		6´´´´		7.50 (br s)	7.50/7.37 (br s)	7.36/7.23 (d, 8.5)	
34	9.77 s	131.50		7´´´´		7.52 (d, 15.9)	6.78 (d, 12.5)	7.45 (d, 16.0)	
35	14.33 s	131.62		8´´´´		6.33 (d, 15.9)	5.83 (d, 12.5)	6.15 (d, 16.0)	
36		157.86		9´´´´					
37		184.59							

[a]δ from TMS (ppm). ^{1}H NMR [400 MHz, J (Hz)] and ^{13}C NMR [100 MHz] data for AC leaves extract (**A**). br (broad signal). Chemical shifts obtained from x (40,41), Y (40,41) and Z (42). All the NMR data were obtained from similar NMR experiments with DMSO-d6 as a solvent.

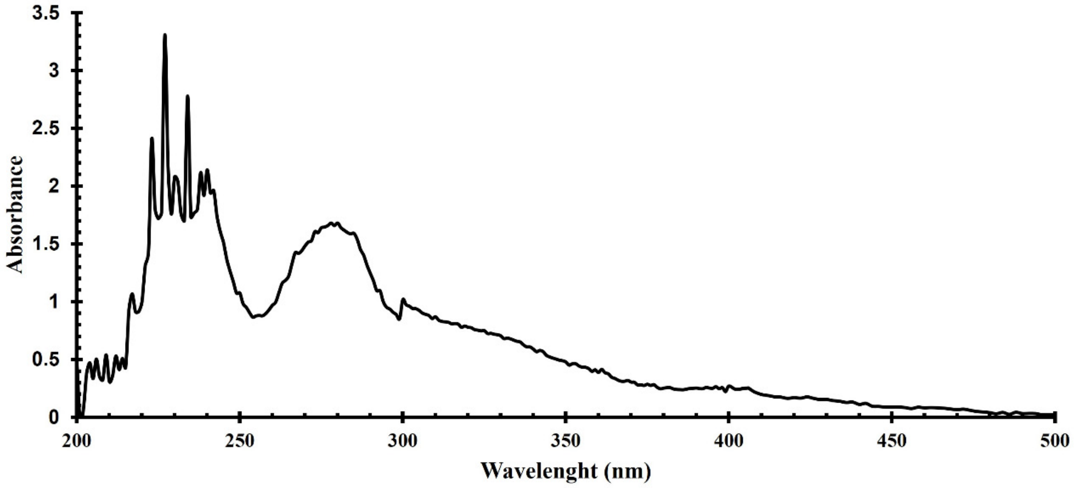

Figure S1. UV spectra recorded in MeOH at 0.1mg/mL.

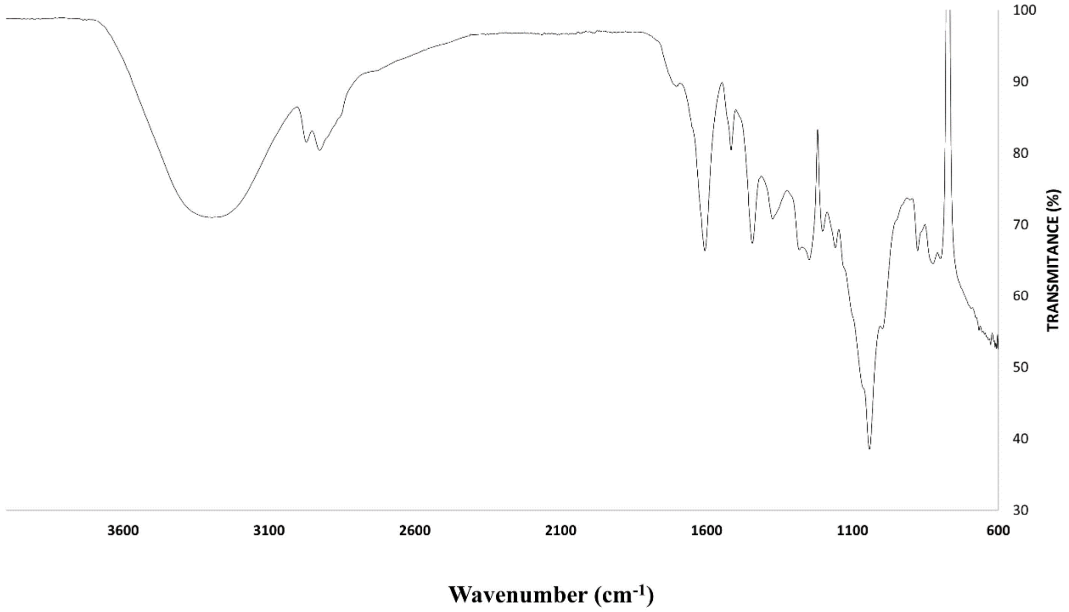

Figure S2. Infrared spectra (wavenumber in cm^{-1}).

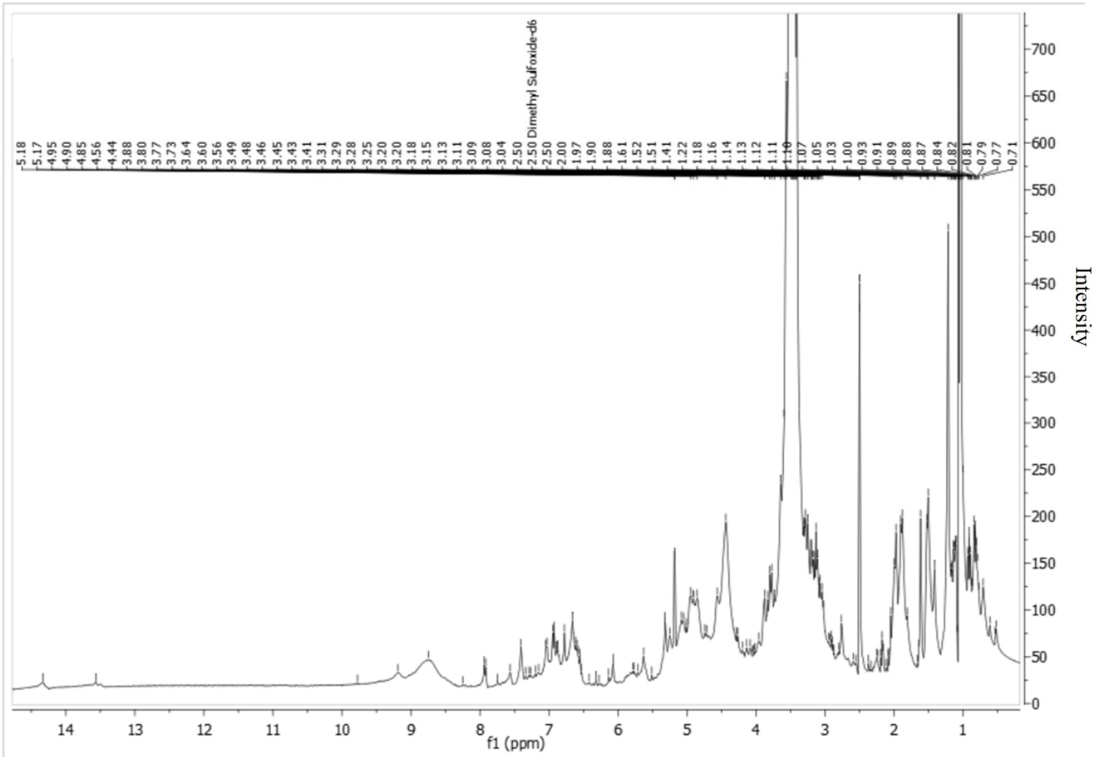

Figure S3. ^{1}H-NMR spectra (400 MHz) of *Averrhoa carambola* extract, recorded in DMSO-d6.

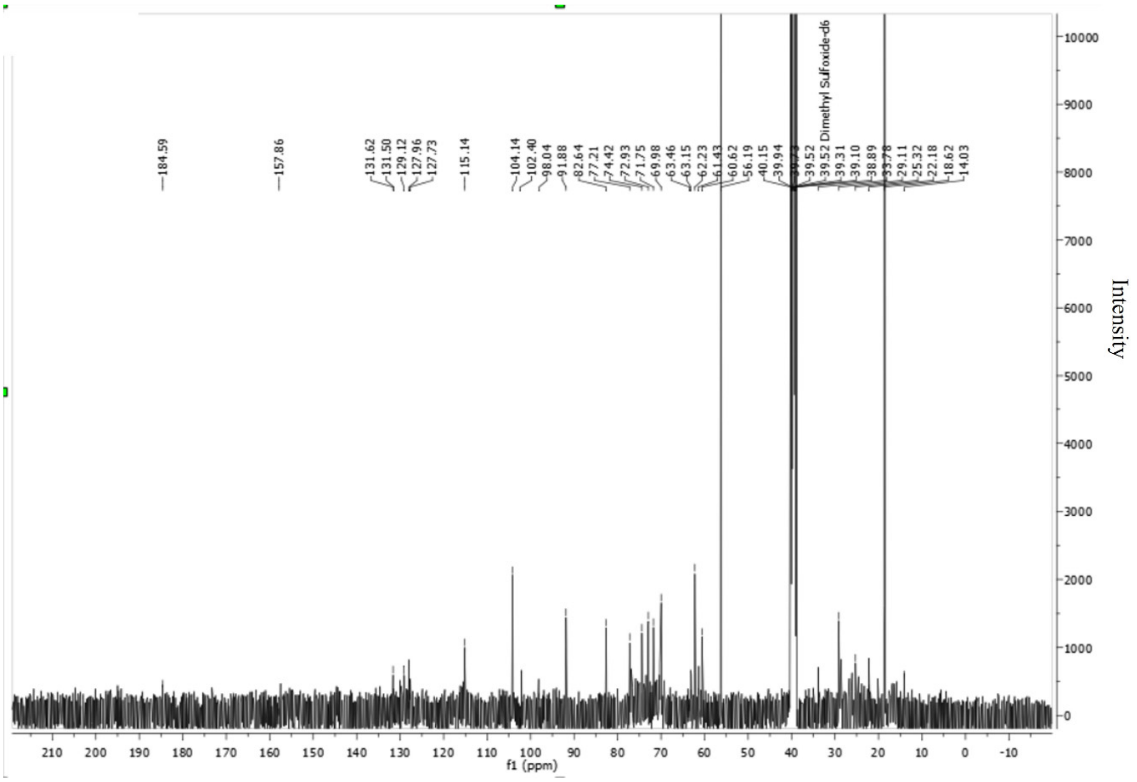

Figure S4. ^{13}C-NMR spectra (100 MHz) of *Averrhoa carambola* extract recorded in DMSO-d6.

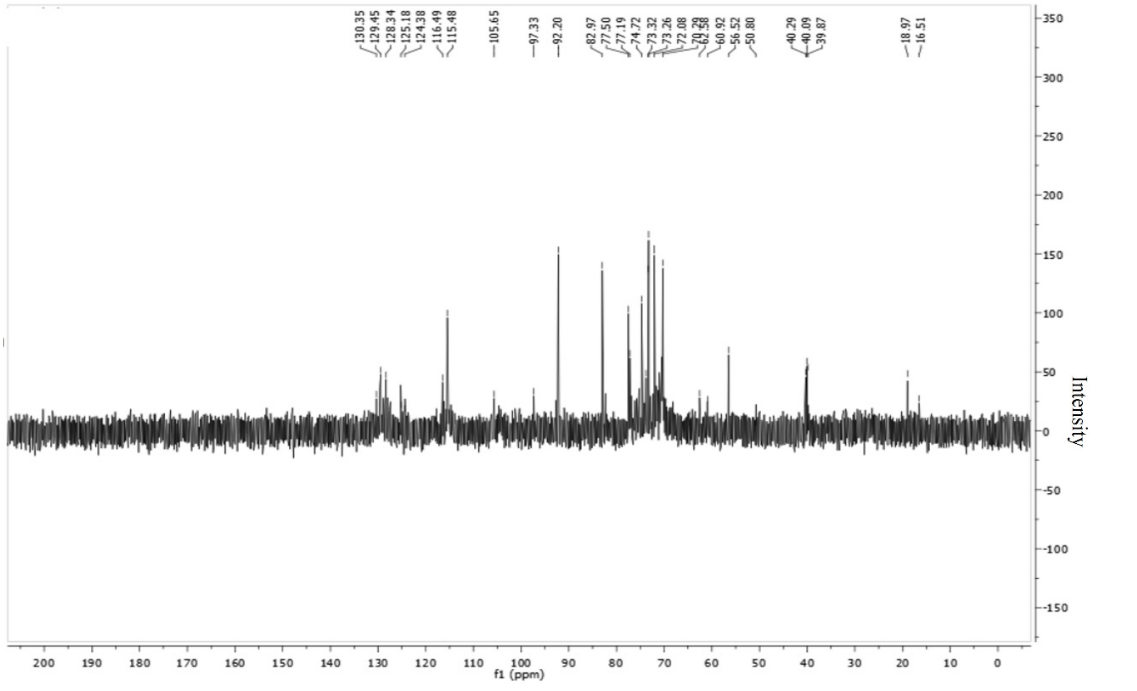

Figure S5. DEPT90 experiment of EE of *Averrhoa carambola*, recorded in DMSO-d6.

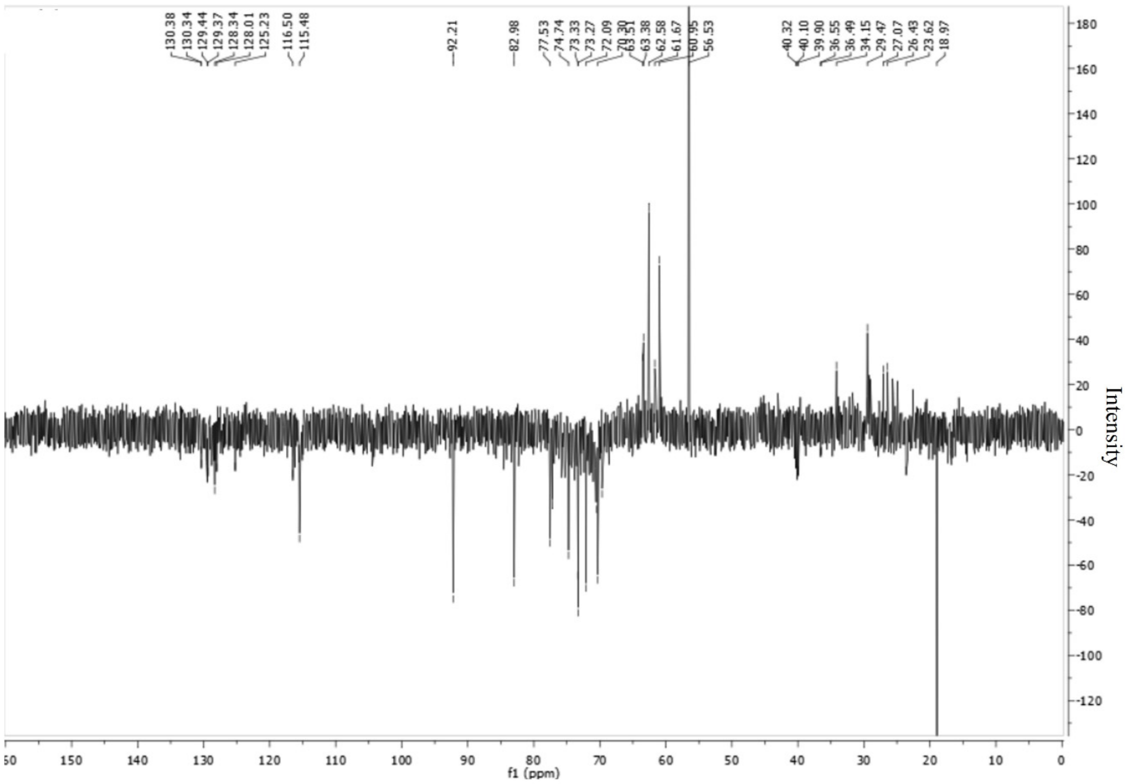

Figure S6. DEPT135 experiment of EE of *Averrhoa carambola*, recorded in DMSO-d6.
